# Health Care Expenses and Financial Hardship Among Medicare Beneficiaries With Functional Disability

**DOI:** 10.1001/jamanetworkopen.2024.17300

**Published:** 2024-06-17

**Authors:** Sungchul Park, Jim P. Stimpson

**Affiliations:** 1 Department of Health Policy and Management, College of Health Science, Korea University, Seoul, Republic of Korea; 2 BK21 FOUR R&E Center for Learning Health Systems, Korea University, Seoul, Republic of Korea; 3 Peter O’Donnell Jr. School of Public Health, The University of Texas Southwestern Medical Center, Dallas

## Abstract

**Importance:**

Medicare beneficiaries with functional disabilities often require more medical care, leading to substantial financial hardship. However, the precise magnitude and sources of this hardship remain unknown.

**Objectives:**

To quantify the financial burden from health care expenses by functional disability levels among Medicare beneficiaries.

**Design, Setting, and Participants:**

This cross-sectional study used data, including demographic and socioeconomic characteristics, health status, and health care use, from a nationally representative sample of Medicare beneficiaries from the 2013 to 2021 Medical Expenditure Panel Survey. Functional disability was measured using 6 questions and categorized into 3 levels: none (no difficulties), moderate (1-2 difficulties), and severe (≥3 difficulties). Data were analyzed from December 2023 to March 2024.

**Main Outcomes and Measures:**

Financial hardship from health care expenses was assessed using objective measures (annual out-of-pocket spending, high financial burden [out-of-pocket spending exceeding 20% of income], and catastrophic financial burden [out-of-pocket spending exceeding 40% of income]) and subjective measures (difficulty paying medical bills and paying medical bills over time). We applied weights to produce results representative of national estimates.

**Results:**

The sample included 31 952 Medicare beneficiaries (mean [SD] age, 71.1 [9.7] years; 54.6% female). In weighted analyses, severe functional disability was associated with a significantly higher financial burden from health care expenses, with out-of-pocket spending reaching $2137 (95% CI, $1943-$2330) annually. This exceeded out-of-pocket spending for those without functional disability by nearly $700 per year ($1468 [95% CI, $1311-$1625]) and for those with moderate functional disability by almost $500 per year ($1673 [95% CI, $1620-$1725]). The primary factors that played a role in this difference were home health care ($399 [95% CI, $145-$651]) and equipment and supplies ($304 [95% CI, $278-$330]). Beneficiaries with severe functional disability experienced significantly higher rates of both high and catastrophic financial burden than those without disability and those with moderate disability (13.2% [12.2%-14.1%] vs 9.1% [95% CI, 8.6%-9.5%] and 9.4% [95% CI, 9.1%-9.7%] for high financial burden, respectively, and 8.9% [95% CI, 7.8%-10.1%] vs 6.4% [95% CI, 6.1%-6.8%] and 6.0% [95% CI, 5.6%-6.4%] for catastrophic financial burden, respectively). Similar associations were observed in subjective financial hardship. For example, 11.8% (95% CI, 10.3%-13.3%) of those with severe functional disability experienced problems paying medical bills, compared with 7.7% (95% CI, 7.6%-7.9%) and 9.3% (95% CI, 9.0%-9.6%) of those without functional disability and those with moderate functional disability, respectively. Notably, there were no significant differences in financial hardship among those with Medicaid based on functional disability levels.

**Conclusions and Relevance:**

In this cross-sectional study of Medicare beneficiaries, those with severe functional disability levels experienced a disproportionate burden from health care costs. However, Medicaid played a pivotal role in reducing the financial strain. Policymakers should explore interventions that effectively relieve the financial burden of health care in this vulnerable population.

## Key Points

**Question:**

What is the financial burden of health care expenses among Medicare beneficiaries with functional disability?

**Findings:**

In this cross-sectional study of 31 952 Medicare beneficiaries, those with severe functional disability had significantly greater out-of-pocket spending on health care annually compared with those with no or moderate functional disability. Spending variation was attributable to home health care and equipment and supplies.

**Meaning:**

Findings of this study suggest that severe functional disability amplifies the financial burden of health care for Medicare beneficiaries and warrant interventions to relieve the financial burden of care.

## Teaser

This cross-sectional study examines health care expenses and financial hardship among Medicare beneficiaries with functional disability.

## Introduction

Functional disability, defined as acquired difficulties in performing daily tasks or maintaining independent living, has become increasingly prevalent with advancing age in the US.^1^ In 2019, 66.1% of US adults aged 65 years and older reported experiencing functional limitations in at least 1 of the 6 core functional domains (seeing, hearing, mobility, communication, cognition, and self-care), with 46.8% indicating substantial difficulties and 19.3% reporting some level of impairment.^2^ This phenomenon often results from a complex interplay of age-related physical and cognitive changes, chronic health conditions, and social factors.^3,4^ Older adults undergo a gradual decrease in physical and cognitive capacities due to the aging process. These functional limitations lead to acute morbidity conditions and further functional losses, culminating in a state of disability that considerably impacts well-being and independence.

Adults with functional disability experience many co-occurring medical, behavioral health, and social challenges, incurring greater health care expenditures.^5,6,7,8,9,10,11,12^ Functional disability is associated with multiple adverse health outcomes, including hospitalization, depression, social isolation, and death.^4,13^ Consequently, total health care expenditures are higher among those with functional disability than those without functional disability, and disproportionately increase with the severity of functional disability. One study reported total annual expenditures of $1934 for those with 0 restrictions in activities of daily living (ADLs), $4540 for those with 1 or 2 restricted ADLs, $7589 for those with 3 or 4 restricted ADLs, and $14 399 for those with 5 or 6 restricted ADLs.^5^ Furthermore, total health care expenditures attributable to functional disability more than doubled over the past decade, rising from $398 billion in 2003 to $868 billion in 2015.^11^ Notably, per-person spending for individuals without disability remained constant, while disability-associated health care expenditure increased from $13 395 in 2003 to $17 431 in 2015.^11^

The additional costs associated with functional disability can impose a disproportionately high financial burden. Adults with disabilities were nearly 3 times more likely to report financial hardship than adults without disabilities.^14^ Moreover, median out-of-pocket spending among adults with disabilities increased substantially from 1996 to 2004, rising by 124% compared with a 62% increase among adults without disabilities.^8^ As persons with disabilities often have unmet basic needs, limited financial assets, and low health insurance literacy,^15,16,17^ the financial burden of health care costs may exacerbate existing disparities, further marginalizing an already socially and economically vulnerable population. This is particularly relevant to Medicare beneficiaries given that the largest portion of disability-associated health care expenditure is covered by the Medicare program.^11^

Despite its importance, there are gaps in our understanding of financial hardship among Medicare beneficiaries with functional disability. First, some studies have used relatively outdated data^8,11^; thus, there is limited evidence of the recent trend in financial hardship associated with functional disability. Second, much of the prior research has relied on self-reported measures, raising concerns about the validity of the findings.^14^ Third, few studies have focused solely on total out-of-pocket spending, leaving the specific sources of this hardship unclear.^8^ Finally, Medicaid may have the potential to alleviate financial hardship among those with functional disability,^18^ but less is known about the specific role of Medicaid in this context.

To address this knowledge gap, we examined the association between the financial burden from health care expenses among Medicare beneficiaries based on functional disability levels. Specifically, we compared objective and subjective financial burden among Medicare beneficiaries by level of functional disability. Also, we performed this analysis for the entire population and subpopulations based on Medicaid status.

## Methods

### Data

This cross-sectional study used data from the 2013 to 2021 Medical Expenditure Panel Survey (MEPS), which is a nationally representative survey of the US noninstitutionalized civilian population. The survey follows the same cohort of households over a 2-year period, collecting information on demographic and socioeconomic characteristics, health status, and health care use. The MEPS is reviewed and approved annually by the Westat Institutional Review Board. In accordance with the Common Rule, this study was exempt from review and the informed consent requirement since it used fully deidentified publicly available data. We adhered to the Strengthening the Reporting of Observational Studies in Epidemiology (STROBE) reporting guideline.

### Sample

We identified Medicare beneficiaries (including those younger than 65 years and those aged 65 years or older) and then categorized the sample by functional disability levels. Specifically, functional disability was determined based on 6 questions assessing difficulties in seeing, hearing, memory or concentration, walking, self-care, and performing errands due to a physical, mental, or emotional condition.^19^ These 6 questions adhered to the established data standard for survey questions on disability codified by the US Department of Health and Human Services.^20^ Questions from the MEPS included the following: Does anyone in the family have any difficulty seeing? Does anyone in the family have any difficulty hearing? Do any of the adults in the family experience confusion or memory loss such that it interferes with daily activities? Does anyone in the family have any difficulty walking, climbing stairs, grasping objects, reaching overhead, lifting, bending or stooping, or standing for long periods? Does anyone in the family receive help or supervision with personal care such as bathing, dressing, or getting around the house? And because of a physical, mental, or emotional condition, do you have difficulty doing errands alone such as visiting a doctor’s office or shopping? If the response was yes, a follow-up question was asked to determine which household members had difficulty. We assigned each question a value of 1 to indicate the presence of a difficulty and 0 to indicate no difficulty. The scores from the 6 questions were then aggregated and used to classify functional disability into 3 levels: none (0 difficulties identified by any of the 6 questions), moderate (1-2 difficulties), and severe (≥3 difficulties).

### Outcome Variables

Our primary outcomes included objective and subjective financial hardship from health care expenses.^21^ Objective financial hardship was assessed using 3 measures: annual out-of-pocket spending (the total amount of health care expenses covered directly by individuals or families), high financial burden (whether out-of-pocket spending exceeded 20% of annual postsubsistence family income [income after accounting for food-related expenses]), and catastrophic financial burden (whether out-of-pocket spending exceeded 40% of annual postsubsistence family income).^22^ To pinpoint the primary source of financial hardship, we analyzed out-of-pocket spending both in aggregate and for 6 specific service categories (inpatient admissions, clinician visits, emergency department [ED] visits, prescription drugs, home health, and equipment and supplies). Out-of-pocket spending was adjusted to 2021 dollars using the Personal Consumption Expenditures Price Index for health care.^23^ Subjective financial hardship was assessed through 2 self-reported measures: family having problems paying medical bills and family paying medical bills over time. We treated out-of-pocket spending as continuous variables while all others were binary. As secondary outcomes, we evaluated binary utilization measures for 6 service categories (inpatient admissions, clinician visits, ED visits, prescription drugs, home health, and equipment and supplies).

### Covariates

To account for differences in sample characteristics by functional disability level, we included age, sex, race and ethnicity, employment status, marital status, education, family income, health insurance (Medicare Advantage, Medicare prescription drug coverage, Medicaid coverage, and any private insurance coverage), US Census region of residence, number of chronic conditions, and health-related quality of life (the Physical Component Summary score and the Mental Component Summary score from the Short Form-12 Health Survey). Race and ethnicity were assessed by self-report within the MEPS dataset and were included to better characterize the study sample. Health insurance categories overlapped as Medicare beneficiaries could have coverage from multiple sources (eg, Medicare Advantage and Medicaid). The Short Form-12 Health Survey score is a standardized health measure scored on a scale of 0 to 100, with higher scores indicating better health.

### Statistical Analysis

We compared sample characteristics of Medicare beneficiaries by functional disability levels. To quantify and compare the financial burden associated with functional disability, we conducted regression models with financial hardship as the outcome variable and functional disability status as the primary independent variable while controlling for individual-level characteristics. Out-of-pocket spending was analyzed using a 2-part model (a logistic regression model to estimate the probability of incurring any positive spending and a generalized linear model with log link and γ family to estimate positive spending values) and binary outcomes were analyzed using linear probability models. To produce interpretable and comparable results, we calculated the mean adjusted values of the outcomes for each group while holding constant all other variables, allowing us to compare the outcome of interest among Medicare beneficiaries across functional disability levels. Furthermore, Medicaid may play a pivotal role in reducing the financial burden. To examine potential variations in financial hardship associated with functional disability among individuals with and without Medicaid, we conducted stratified analyses for those dual-eligible for Medicare and Medicaid and those dual-ineligible for Medicare and Medicaid. For all analyses, we included year fixed effects and used survey weights to generate nationally representative estimates. Data were analyzed from December 2023 to March 2024 using Stata, version 16.1 (StataCorp LLC). Reported *P* values were 2-sided, and *P* < .05 represented statistical significance.

## Results

### Sample Characteristics

Our final sample consisted of 31 952 Medicare beneficiaries (mean [SD] age, 71.1 [9.7] years; 56.0% female and 44.0% male), including 17 286 with no functional disability, 10 562 with moderate functional disability, and 4104 with severe functional disability. We found differences in sample characteristics by functional disability levels. Based on weighted estimates, individuals with functional disability compared with those without functional disability were more likely to be younger than 65 years or older than 85 years, have Medicaid coverage, have a greater number of chronic conditions, have lower Physical Component Summary and Mental Component Summary scores on the Short Form-12 Health Survey, and they were less likely to be employed, be married, have an advanced degree, have higher family income, and have private health insurance coverage (Table). Notably, these differences were more pronounced among individuals with severe functional disability than those with moderate functional disability. Approximately 7% of Medicare beneficiaries with no functional disability had Medicaid, but the proportion with Medicaid doubled among those with moderate functional disability (14%) and doubled again among those with severe functional disability (28%). Commensurately, half of beneficiaries with severe functional disability and 37% of those with moderate functional disability reported a family income less than 200% below the federal poverty level.

**Table.** Sample Characteristics of Medicare Beneficiaries by Functional Disability Level, 2013-2021^a^

Characteristic	No. (weighted %)		
	No functional disability (n = 17 286)	Moderate functional disability (n = 10 562)	Severe functional disability (n = 4104)
Age, y			
<65	1318 (6.6)	1945 (17.1)	1005 (23.3)
65-74	10 751 (63.1)	4394 (41.6)	1114 (25.2)
75-84	4400 (25.8)	3018 (29.7)	1088 (26.8)
≥85	817 (4.6)	1205 (11.6)	897 (24.7)
Sex			
Female	9557 (54.6)	5779 (53.1)	2557 (60.4)
Male	7729 (45.4)	4783 (46.9)	1547 (39.6)
Race and ethnicity			
Hispanic	1886 (7.3)	1114 (7.0)	676 (10.2)
Non-Hispanic Asian	802 (4.2)	249 (2.1)	116 (2.8)
Non-Hispanic Black	2464 (9.2)	1692 (9.8)	706 (11.2)
Non-Hispanic White	11 811 (77.5)	7177 (77.9)	2442 (72.1)
Non-Hispanic other or multiple^b^	323 (1.8)	330 (3.3)	164 (3.7)
Employed	4093 (25.4)	1328 (14.0)	135 (3.7)
Married	9777 (61.1)	4675 (49.6)	1241 (33.8)
Education			
High school	1978 (7.8)	1735 (12.6)	1025 (20.4)
College graduate	7198 (41.5)	5043 (48.0)	1861 (47.1)
Advanced degree	7152 (44.5)	3084 (32.0)	870 (24.0)
Family income			
<200% of FPL	5251 (24.3)	4727 (37.4)	2436 (52.5)
200%-399% of FPL	4818 (27.0)	2909 (28.0)	1008 (26.7)
≥400% of FPL	7217 (48.7)	2926 (34.7)	660 (20.8)
Health insurance coverage^c^			
Medicare Advantage	7118 (39.6)	4226 (38.5)	1670 (39.4)
Medicare prescription drug	10 500 (59.7)	6507 (60.8)	2627 (62.9)
Medicaid	1731 (6.7)	2061 (14.1)	1472 (28.9)
Private	7741 (49.7)	3690 (40.2)	1045 (31.1)
US Census region			
Northeast	2993 (18.2)	1622 (16.4)	619 (16.5)
Midwest	3591 (21.7)	2410 (23.0)	832 (21.2)
South	6491 (37.6)	4285 (40.3)	1756 (41.2)
West	4211 (22.5)	2245 (20.3)	897 (21.1)
No. of chronic conditions			
0	7258 (42.2)	2770 (26.5)	652 (16.1)
1-2	8392 (48.3)	5533 (51.9)	1999 (48.4)
3-5	1597 (9.3)	2130 (20.2)	1292 (31.0)
≥6	39 (0.2)	129 (1.3)	161 (4.4)
SF-12 health-related quality of life, mean (SD)^d^			
Physical Component score	47.6 (9.5)	37.2 (11.7)	27.8 (9.1)
Mental Component score	54.2 (7.7)	50.6 (10.5)	43.4 (12.1)

Abbreviations: FPL; federal poverty level, SF-12; Short Form-12 Health Survey.

^a^ Functional disability level was measured using 6 questions assessing difficulties and categorized into 3 levels: none (no difficulties), moderate (1-2 difficulties), and severe (≥3 difficulties). Survey weights were used to adjust sample characteristics to be representative of the US population.

^b^ Other includes individuals who did not self-identify as one of the specified subpopulation groups.

^c^ Health insurance categories overlapped as Medicare beneficiaries could have coverage from multiple sources.

^d^ The Short Form-12 Health Survey is a standardized health measure scored on a scale of 0 to 100, with higher scores indicating better health.

### Financial Hardship by Functional Disability Level

**Figure 1.**
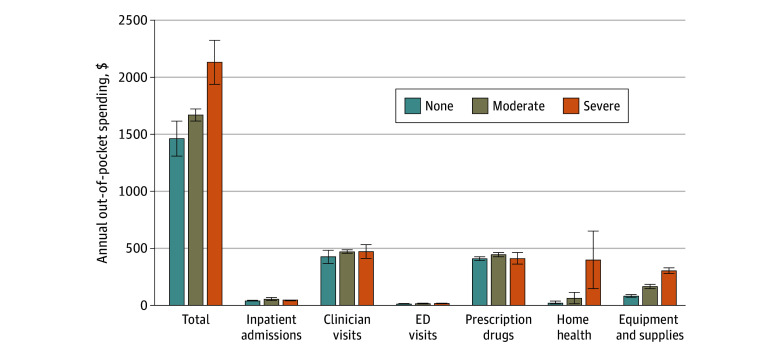
Annual Out-of-Pocket Spending Among Medicare Beneficiaries by Functional Disability Level From 2013 to 2021 Data adjusted to 2021 dollars. Functional disability was classified as none (no difficulties in 6 areas assessed), moderate (1-2 difficulties), or severe (≥3 difficulties). Error bars indicate 95% CIs. ED indicates emergency department.

Functional disability was associated with greater objective financial hardship from health care expenses among Medicare beneficiaries, but this association was most pronounced among individuals with severe functional disability (Figure 1; eTable 1 in Supplement 1). Beneficiaries with severe functional disability were significantly more likely to report higher financial burden from health care expenses, with out-of-pocket spending reaching $2137 (95% CI, $1943-$2331). This financial burden was nearly $700 more than for those without functional disability ($1468 [95% CI, $1311-$1625]) and $500 more than for those with moderate functional disability ($1673 [95% CI, $1620-$1725]). There were significantly higher rates of high and catastrophic financial burden among beneficiaries with severe functional disability than those without disability and those with moderate disability (13.2% [95% CI, 12.2%-14.1%] vs 9.1% [95% CI, 8.6%-9.5%] and 9.4% [95% CI, 9.1%-9.7%], respectively, for high financial burden and 8.9% [95% CI, 7.8%-10.1%] vs 6.4% [95% CI, 6.1%-6.8%] and 6.0% [95% CI, 5.6%-6.4%] for catastrophic financial burden, respectively). Notably, the factors that played a role in the difference in out-of-pocket spending were home health care ($21 [95% CI, $6-$37], $63 [95% CI, $10-$116], and $399 [95% CI, $145-$651] for beneficiaries with no, moderate, and severe functional disabilities, respectively) and equipment and supplies ($82 [95% CI, $70-$93], $166 [95% CI, $148-$182], and $304 [95% CI, $278-$330] for those with no, moderate, and severe functional disabilities, respectively). However, there were no or only small differences in out-of-pocket spending for inpatient admissions, clinician visits, ED visits, and prescription drugs across functional disability levels.

**Figure 2.**
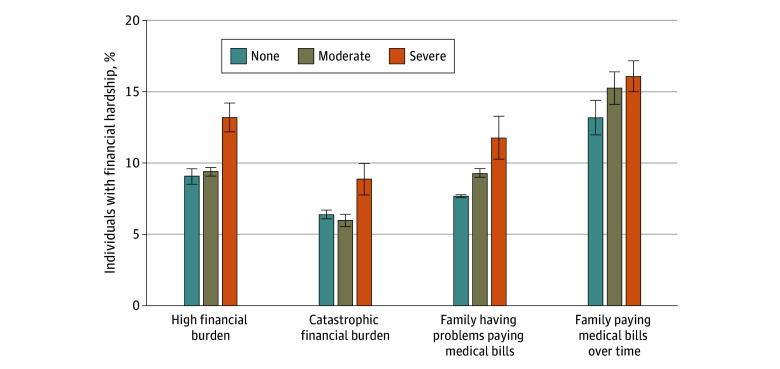
Financial Hardship Among Medicare Beneficiaries by Functional Disability Level From 2013 to 2021 Functional disability was classified as none (no difficulties in 6 areas assessed), moderate (1-2 difficulties), or severe (≥3 difficulties). High financial burden indicates out-of-pocket spending exceeding 20% of annual postsubsistence family income (income after accounting for food-related expenses). Catastrophic financial burden indicates out-of-pocket spending exceeding 40% of annual postsubsistence family income. Error bars indicate 95% CIs.

Similar associations were observed in subjective financial burden (Figure 2; eTable 1 in Supplement 1). In all, 11.8% (95% CI, 10.3%-13.3%) of Medicare beneficiaries with severe functional disability experienced problems paying medical bills compared with 7.7% (95% CI, 7.6%-7.9%) of those without functional disability and 9.3% (95% CI, 9.0%-9.6%) of those with moderate functional disability. Also, 16.1% (95% CI, 15.0%-17.2%) of beneficiaries with severe functional disability reported difficulty paying medical bills over time compared with 13.2% (95% CI, 12.0%-14.4%) of those without functional disability and 15.3% (95% CI, 14.2%-16.4%] of those with moderate functional disability.

### Health Care Utilization by Functional Disability Level

**Figure 3.**
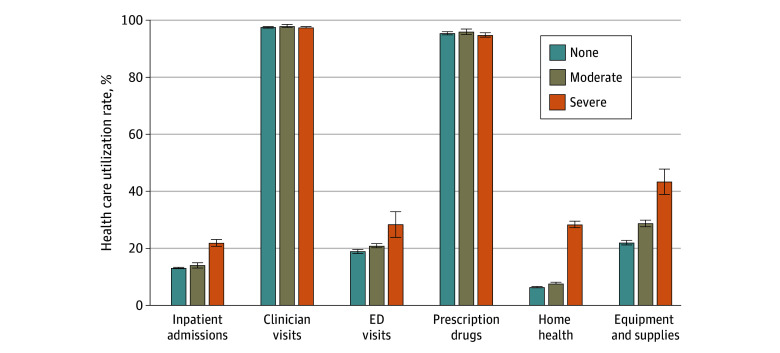
Health Care Use Among Medicare Beneficiaries by Functional Disability Level From 2013 to 2021 Functional disability was classified as none (no difficulties in 6 areas assessed), moderate (1-2 difficulties), or severe (≥3 difficulties). Error bars indicate 95% CIs. ED indicates emergency department.

Severe functional disability was associated with higher health care utilization rates compared with no functional disability and moderate functional disability (Figure 3; eTable 2 in Supplement 1). Notable differences were seen in home health care (6.4% [95% CI, 6.2%-6.5%], 7.7% [95% CI, 7.4%-8.0%], and 28.4% [95% CI, 27.2%-29.6%] for those with no, moderate, and severe functional disability levels, respectively) and equipment and supplies (22.0% [95% CI, 21.2%-22.7%], 28.8% [95% CI, 27.7%-29.9%], and 43.4% [95% CI, 39.0%-47.8%] for those with no, moderate, and severe functional disability levels, respectively). Also, inpatient admissions and ED visits were more prevalent among those with severe functional disability, but the differences were smaller. There were no or only small differences in clinician visits and prescription drugs across functional disability levels.

### Financial Hardship by Medicaid Dual Eligibility Status

**Figure 4.**
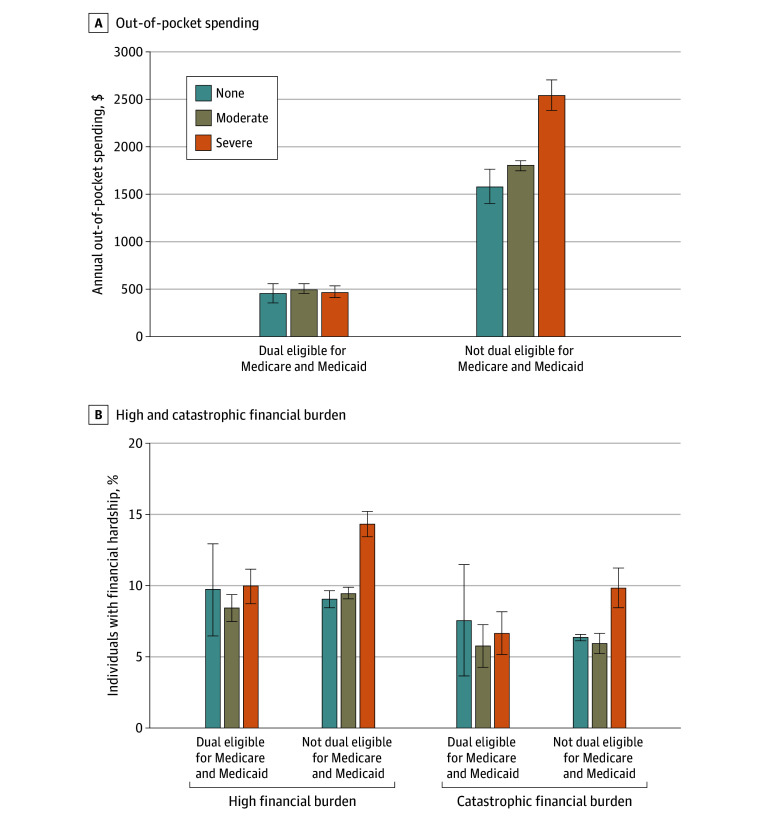
Differences in Objective Financial Hardship Among Medicare Beneficiaries With and Without Dual Medicare and Medicaid Eligibility by Functional Disability Level From 2013 to 2021 Data adjusted to 2021 dollars. Functional disability was classified as none (no difficulties in 6 areas assessed), moderate (1-2 difficulties), or severe (≥3 difficulties). High financial burden indicates out-of-pocket spending exceeding 20% of annual postsubsistence family income (income after accounting for food-related expenses). Catastrophic financial burden indicates out-of-pocket spending exceeding 40% of annual postsubsistence family income. Error bars indicate 95% CIs.

Financial hardship among those with dual eligibility for Medicare and Medicaid did not significantly vary by functional disability levels, but there were significant differences in financial hardship among those without dual eligibility for Medicare and Medicaid based on functional disability levels (Figure 4; eTables 3 and 4 in Supplement 1). Among those with dual eligibility, there were no significant differences in out-of-pocket spending based on functional disability levels ($459 [95% CI, $363-$555], $507 [95% CI, $471-$543], and $473 [95% CI, $417-$529] for those with no, moderate, and severe functional disability levels, respectively). Additionally, there were no significant differences in the rates of high and catastrophic financial burden by functional disability levels. However, among those without dual eligibility, there were significant differences in out-of-pocket spending based on functional disability levels ($1587 [95% CI, $1413-$1760], $1813 [95% CI, $1767-$1859], and $2555 [95% CI, $2397-$2712] for those with no, moderate, and severe functional disability levels, respectively). Consequently, being a non–dual-eligible Medicare beneficiary with severe functional disability was associated with significantly higher rates of both high and catastrophic financial burden than for dual-eligible Medicare beneficiaries without functional disability and those with moderate functional disability (11.5% [95% CI, 9.7%-13.2%] vs 7.1% [95% CI, 7.0%-7.2%] and 8.6% [95% CI, 8.4%-8.9%] for high financial burden, respectively, and 16.0% [95% CI, 15.2%-16.7%] vs 13.1% [95% CI, 12.0%-14.3%] and 15.3% [95% CI, 14.1%-16.4%] for catastrophic financial burden, respectively).

## Discussion

This analysis of a nationally representative sample found that functional disability was associated with greater objective and subjective financial hardship from health care expenses among Medicare beneficiaries, but this association was most pronounced among those with severe functional disability. Expenses related to home health care and equipment and supplies were primary factors in the financial burden observed. Furthermore, financial hardship among those with dual eligibility for Medicare and Medicaid was not associated with functional disability levels. However, not having dual eligibility for Medicare and Medicaid was associated with considerably greater financial hardship as the functional disability level increased.

Our findings are consistent with prior research that reported a substantial level of financial hardship among Medicare beneficiaries with functional disabilities.^8,11,14^ This financial hardship was most pronounced among those with severe functional disability, primarily attributable to expenses related to home health care and equipment and supplies. This result aligns with prior research indicating a disproportionate increase in the cost of home health care among Medicare beneficiaries with severe functional disability, surpassing the cost of outpatient visits.^5^ These findings suggest that Medicare beneficiaries with severe functional disability have greater needs for home health services. Within the Medicare program, home health services consist of skilled nursing, physical therapy, occupational therapy, speech therapy, aide services, and medical social work provided in the home. Some equipment and supplies, such as durable medical equipment or medical supplies, are provided with home health services. Indeed, our analysis revealed a greater use of home health care and equipment and supplies among those with severe functional disability. Given that home-based care and equipment assistance play a key role in reducing disability,^24,25^ ensuring access to these services for this population becomes imperative.

This study’s findings underscore the important role that Medicaid plays in mitigating financial challenges for Medicare beneficiaries with functional disabilities. The high financial burden associated with functional disability may, in part, result from a high degree of co-occurring medical, behavioral health, and social challenges.^8^ However, some of the financial challenges may stem from limited Medicare coverage. The Medicare program covers a variety of acute and postacute care services, including inpatient hospital stays, skilled nursing care, hospice, and some home health care but has limited coverage of disabled conditions that require services including long-term care, home health care, and durable medical equipment. Consequently, Medicare beneficiaries with functional disabilities experience challenges in accessing care and often experience unmet need for care, leading to preventable health care use.^26,27,28,29^

Addressing the financial challenges faced by Medicare beneficiaries with functional disabilities could be alleviated through state and federal policies aimed at expanding coverage for home and community-based services (HCBS).^30,31^ As HCBS programs are administered through Medicaid and eligibility is based on financial status and certain physical and cognitive limitations, these programs are a viable policy avenue to support those with functional disabilities. Indeed, we found that approximately 7% of Medicare beneficiaries with no functional disability had Medicaid, but the proportion with Medicaid doubled among those with moderate functional disability (14%) and doubled again among those with severe functional disability (28%). Commensurately, half of beneficiaries with severe functional disability and 37% of those with moderate functional disability reported a family income less than 200% below the federal poverty level. Evidence suggests that the use of HCBS substantially increased among those with functional disabilities after state expansions through Section 1915(c) Medicaid waivers.^32,33^ Despite the increase in HCBS use, the Medicaid programs achieved a net savings, primarily due to a decrease in the use of nursing homes.^34^ However, not all Medicare beneficiaries are eligible for HCBS programs. Therefore, individuals with a functional disability who are currently ineligible for Medicaid due to a state’s financial eligibility requirements could benefit from an expansion of Medicaid eligibility. Drawing insights from the lessons learned from Patient Protection and Affordable Care Act incentive programs, the American Rescue Plan Act introduced a 1-year 10% increase in the Federal Medical Assistance Percentage for Medicaid HCBS programs.^35^ This incentive was designed to be more accessible for states to implement. Early evidence indicates that all 50 states have submitted plans to enhance the HCBS workforce and quality.^36^ Thus, the American Rescue Plan Act incentive may prove successful as a short-term federal policy to improve HCBS access.

### Limitations

This study has some limitations. First, there is no standard criterion for subjective financial hardship or objective financial hardship. Second, the sample was limited to the noninstitutionalized US population, excluding people experiencing incarceration, nursing home residents, and those in residential treatment. Third, while we used a measure of functional disability developed and assessed in prior research, the measures are self-reported, and respondents may underreport mental health problems due to stigma or privacy concerns. Fourth, we included a range of demographic, socioeconomic, and health characteristics to control for sample characteristics, but unobserved differences in patient factors may still exist. Fifth, those with functional disability report limited access to care and unmet need for care, indicating that the financial burden of care may be underestimated. Finally, our analysis is observational, and we are unable to make causal interpretations based on our results.

## Conclusions

This cross-sectional study found that functional disability was associated with increased financial hardship from health care expenses among Medicare beneficiaries, and this association was most prominent among those with severe functional disability levels. The primary factors in this financial burden were expenses related to home health care and equipment and supplies. This research underscores the important role that Medicaid plays in mitigating financial challenges for those with functional disabilities. To effectively address the financial strain experienced by Medicare beneficiaries with functional disabilities, the implementation of policy interventions is crucial. An essential step is expanding coverage for essential services, providing comprehensive support for this population.

## Notes

Supplement 1. **eTable 1.** Financial Hardship Among Medicare Beneficiaries by Functional Disability Level, 2013 to 2021 MEPS **eTable 2.** Health Care Utilization Among Medicare Beneficiaries by Functional Disability Level, 2013 to 2021 MEPS **eTable 3.** Financial Hardship and Health Care Utilization Among Medicare Beneficiaries With Medicaid by Functional Disability Level, 2013 to 2021 MEPS **eTable 4.** Financial Hardship and Health Care Utilization Among Medicare Beneficiaries Without Medicaid by Functional Disability Level, 2013 to 2021 MEPS

Supplement 2.
Data Sharing Statement
