# Supplementary material for: Health Care Expenses and Financial Hardship Among Medicare Beneficiaries With Functional Disability
Source: JAMA Netw Open. 2024 Jun 17;7(6):e2417300. doi: 10.1001/jamanetworkopen.2024.17300 (PMC11184460; doi:10.1001/jamanetworkopen.2024.17300)
Supplement: Supplement 1. — eTable 1. Financial Hardship Among Medicare Beneficiaries by Functional Disability Level, 2013 to 2021 MEPS eTable 2. Health Care Utilization Among Medicare Beneficiaries by Functional Disability Level, 2013 to 2021 MEPS eTable 3. Financial Hardship and Health Care Utilization Among Medicare Beneficiaries With Medicaid by Functional Disability Level, 2013 to 2021 MEPS eTable 4. Financial Hardship and Health Care Utilization Among Medicare Beneficiaries Without Medicaid by Functional Disability Level, 2013 to 2021 MEPS [file jamanetwopen-e2417300-s001.pdf]

Supplemental Online Content

Park S, Stimpson JP. Health care expenses and financial hardship among Medicare beneficiaries with functional disability. *JAMA Netw Open.* 2024;7(6):e2417300. doi:10.1001/jamanetworkopen.2024.17300

eTable 1. Financial Hardship Among Medicare Beneficiaries by Functional Disability Level, 2013 to 2021 MEPS

eTable 2. Health Care Utilization Among Medicare Beneficiaries by Functional Disability Level, 2013 to 2021 MEPS

eTable 3. Financial Hardship and Health Care Utilization Among Medicare Beneficiaries With Medicaid by Functional Disability Level, 2013 to 2021 MEPS

eTable 4. Financial Hardship and Health Care Utilization Among Medicare Beneficiaries Without Medicaid by Functional Disability Level, 2013 to 2021 MEPS

This supplemental material has been provided by the authors to give readers additional information about their work.

eTable 1. Financial Hardship Among Medicare Beneficiaries by Functional Disability Level, 2013 to 2021 MEPS

Outcomes	Adjusted estimates (95% CI)		
	No disability (N=17286)	Moderate disability (N=10562)	Severe disability (N=4104)
Objective financial hardship			
OOP spending, $			
Total	1468.6 (1311.4, 1625.7)	1673 (1620.9, 1725.1)	2137.1 (1943.3, 2330.9)
Inpatient admissions	36.1 (29.4, 42.8)	53.5 (40.4, 66.7)	44.3 (42.9, 45.7)
Provider visits	427.1 (369.9, 484.2)	472.2 (456.9, 487.5)	472.9 (414.1, 531.8)
ER visits	11.4 (9.3, 13.6)	15.2 (12.9, 17.5)	17.6 (12.6, 22.6)
Prescription drugs	409.5 (395.2, 423.9)	446.3 (428, 464.7)	412.3 (361.8, 462.8)
Home health	21.9 (6.7, 37.2)	63.5 (10.2, 116.8)	398.6 (145.6, 651.6)
Equipment and supplies	82.1 (70.4, 93.8)	165.7 (148.6, 182.8)	304.3 (278.2, 330.4)
High burden, %	9.1 (8.6, 9.5)	9.4 (9.1, 9.7)	13.2 (12.2, 14.1)
Catastrophic burden, %	6.4 (6.1, 6.8)	6 (5.6, 6.4)	8.9 (7.8, 10.1)
Subjective financial hardship			
Family having problems paying medical bills, %	7.7 (7.6, 7.9)	9.3 (9, 9.6)	11.8 (10.3, 13.3)
Family paying medical bills over time, %	13.2 (12, 14.4)	15.3 (14.2, 16.4)	16.1 (15, 17.2)

eTable 2. Health Care Utilization Among Medicare Beneficiaries by Functional Disability Level, 2013 to 2021 MEPS

	Adjusted estimates (95% CI)		
Outcomes	No disability (N=17286)	Moderate disability (N=10562)	Severe disability (N=4104)
Health care utilization (use or not), %			
Inpatient admissions	13.1 (13, 13.3)	14.0 (13.1, 14.8)	21.9 (20.7, 23.1)
Provider visits	97.6 (97.4, 97.9)	98.1 (97.6, 98.7)	97.5 (97.3, 97.8)
ER visits	19.0 (18.2, 19.7)	20.9 (20.2, 21.7)	28.4 (23.9, 32.9)
Prescription drugs	95.6 (95.1, 96.1)	96.1 (95.2, 96.9)	94.9 (94.1, 95.7)
Home health	6.4 (6.2, 6.5)	7.7 (7.4, 8.0)	28.4 (27.2, 29.6)
Equipment and supplies	22.0 (21.2, 22.7)	28.8 (27.7, 29.9)	43.4 (39.0, 47.8)

eTable 3. Financial Hardship and Health Care Utilization Among Medicare Beneficiaries With Medicaid by Functional Disability Level, 2013 to 2021 MEPS

	Adjusted estimates (95% CI)		
Outcomes	No disability (N=1731)	Moderate disability (N=2061)	Severe disability (N=1472)
Financial hardship			
Objective financial hardship			
OOP spending, $			
Total	459.7 (363.8, 555.6)	507.7 (471.5, 543.9)	473.2 (417.1, 529.4)
Inpatient admissions	47.5 (4.9, 90.2)	25.4 (4.5, 46.2)	5.9 (-1, 12.7)
Provider visits	140.5 (89, 192.1)	132.4 (95.8, 169)	99.5 (66.3, 132.7)
ER visits	5.1 (3.3, 6.8)	3.9 (3.5, 4.2)	2.8 (2.3, 3.3)
Prescription drugs	168.6 (138.6, 198.5)	192.6 (173.6, 211.7)	129.7 (107.5, 151.8)
Home health	7.9 (-0.2, 15.9)	5.5 (-2.7, 13.7)	20.2 (-2.5, 42.9)
Equipment and supplies	24.1 (12.7, 35.6)	61.9 (54.4, 69.3)	131.4 (111.7, 151.1)
High burden, %	9.8 (6.6, 12.9)	8.5 (7.6, 9.3)	10 (8.8, 11.1)
Catastrophic burden, %	7.6 (3.7, 11.6)	5.8 (4.3, 7.3)	6.7 (5.2, 8.1)
Subjective financial hardship			
Family having problems paying medical bills, %	12.2 (11.7, 12.7)	14.4 (12.3, 16.6)	15.9 (13.8, 18)
Family paying medical bills over time, %	13.3 (12.2, 14.4)	15.1 (13.6, 16.6)	17.2 (14.4, 20)
Health care utilization (use or not), %			
Inpatient admissions	18.9 (18.2, 19.7)	20.1 (17.5, 22.6)	24.5 (18.2, 30.8)
Provider visits	94.6 (93.3, 95.8)	95.7 (94.4, 97)	94.2 (91.3, 97.1)
ER visits	30.9 (28.9, 32.9)	32.8 (26.9, 38.7)	38.1 (36.2, 39.9)
Prescription drugs	96.9 (96, 97.8)	96.9 (96.2, 97.6)	96.6 (95.8, 97.5)
Home health	15.5 (12.2, 18.9)	22.7 (21.4, 24)	44 (37, 51)
Equipment and supplies	22.5 (18.7, 26.2)	30.3 (28.9, 31.6)	45.2 (44.5, 45.8)

eTable 4. Financial Hardship and Health Care Utilization Among Medicare Beneficiaries Without Medicaid by Functional Disability Level, 2013 to 2021 MEPS

	Adjusted estimates (95% CI)		
Outcomes	No disability (N=15555)	Moderate disability (N=8501)	Severe disability (N=2632)
Financial hardship			
Objective financial hardship			
OOP spending, $			
Total	1587.1 (1413.7, 1760.6)	1813.3 (1767.3, 1859.4)	2555.3 (2397.8, 2712.7)
Inpatient admissions	38.4 (31.7, 45)	57.8 (44.7, 70.9)	52.4 (49.6, 55.1)
Provider visits	463.4 (399, 527.8)	516.5 (497.4, 535.6)	545.2 (485.3, 605.1)
ER visits	12.2 (9.6, 14.8)	17.1 (14.7, 19.6)	20.1 (14.1, 26.1)
Prescription drugs	438.1 (419, 457.1)	478.4 (463.1, 493.8)	481.5 (430.2, 532.8)
Home health	25.5 (6.8, 44.3)	69.9 (12.3, 127.5)	534.2 (80.1, 988.2)
Equipment and supplies	90.3 (76.6, 104)	181.8 (162.4, 201.2)	314.3 (289, 339.6)
High burden, %	9.1 (8.5, 9.7)	9.5 (9.1, 9.9)	14.4 (13.5, 15.4)
Catastrophic burden, %	6.4 (6.2, 6.6)	6 (5.3, 6.7)	9.9 (8.5, 11.4)
Subjective financial hardship			
Family having problems paying medical bills, %	7.1 (7, 7.2)	8.6 (8.4, 8.9)	11.5 (9.7, 13.2)
Family paying medical bills over time, %	13.1 (12, 14.3)	15.3 (14.1, 16.4)	16 (15.2, 16.7)
Health care utilization (use or not), %			
Inpatient admissions	14 (13.8, 14.2)	14.7 (13.8, 15.6)	20.3 (18.8, 21.9)
Provider visits	97.4 (97.1, 97.6)	98 (97.7, 98.3)	97.1 (96.6, 97.6)
ER visits	19.9 (19.7, 20)	22.3 (21.5, 23.1)	29 (26, 31.9)
Prescription drugs	95.7 (95.1, 96.3)	96.4 (95.8, 97)	94.9 (94.1, 95.7)
Home health	7 (6.8, 7.2)	8.6 (8.3, 9)	26.2 (25, 27.4)
Equipment and supplies	22.1 (21.1, 23.1)	29 (27.8, 30.1)	42.4 (38.3, 46.4)
