# Supplementary material for: Health Care Expenses and Financial Hardship Among Medicare Beneficiaries With Functional Disability
Source: JAMA Netw Open. 2024 Jun 17;7(6):e2417300. doi: 10.1001/jamanetworkopen.2024.17300 (PMC11184460; doi:10.1001/jamanetworkopen.2024.17300)
Supplement: Supplement 2. — Data Sharing Statement [file jamanetwopen-e2417300-s002.pdf]

Data Sharing Statement

Park. Health Care Expenses and Financial Hardship Among Medicare Beneficiaries With Functional Disability. *JAMA Netw Open*. Published June 17, 2024.
doi:10.1001/jamanetworkopen.2024.17300

Data
Data available: No

Additional Information
Explanation for why data not available: The data is publicly available.
